# Diagnosis and Management of the Largest Documented Appendicolith in Literature: A Case Report

**DOI:** 10.7759/cureus.54353

**Published:** 2024-02-17

**Authors:** Juliane Hennenberg, Tobias Zott, Vanessa Berger-Kulemann, Ana-Iris Schiefer, Ivan Kristo

**Affiliations:** 1 Radiology, Medical University of Vienna, Vienna, AUT; 2 General Surgery, Medical University of Vienna, Vienna, AUT; 3 Pathology, Medical University of Vienna, Vienna, AUT

**Keywords:** ultrasound-guided, acute appendicitis, appendicitis treatment, emergent general surgery, general radiology, giant appendicolith

## Abstract

This case report depicts the diagnosis and management of the largest documented appendicolith found in the medical literature so far, measuring 4.5 cm. A 44-year-old male patient presented with a distended abdomen, right lower quadrant (RLQ) pain, constipation, and the inability to consume solid food. Laboratory tests revealed leukocytosis and elevated C-reactive protein (CRP) levels. Abdominal X-rays showed a densely calcified structure in the right lower quadrant, and further imaging confirmed the diagnosis of appendicolithiasis. The surgical indication for appendectomy was determined, and an open surgical procedure was performed due to the severity of inflammation, minimal perforation, and extensive adhesions. The surgically removed appendix with the appendicolith was analyzed histologically, confirming appendicolithiasis, periappendicitis, perforation, and serositis. The patient was discharged in stable condition after postoperative management. Giant appendicoliths are rare and associated with an increased risk of complications. Diagnosis is typically clinical but can be enhanced by imaging modalities.

## Introduction

Giant appendicoliths (>2 cm) are rare and often surgically removed due to the increased risk of complications such as perforation or abscess [1,2]. They are more prevalent in children and young adults, particularly in males, and with the presence of a retrocecal appendix [3-5]. The occurrence of appendicoliths in cases of appendicitis varies, ranging from 10% to 55% [4,6,7]. Appendicoliths can be composed of stool (fecalith) and mineral deposits (calculi). There are only a few appendicoliths described over 3 cm [4,5]; the largest appendicolith reported in the literature as of our research measures 3.5 cm and was described in 2005 [8,9]. Migrating appendicoliths can be a rare late-stage complication [10].

## Case presentation

**Figure 1:**
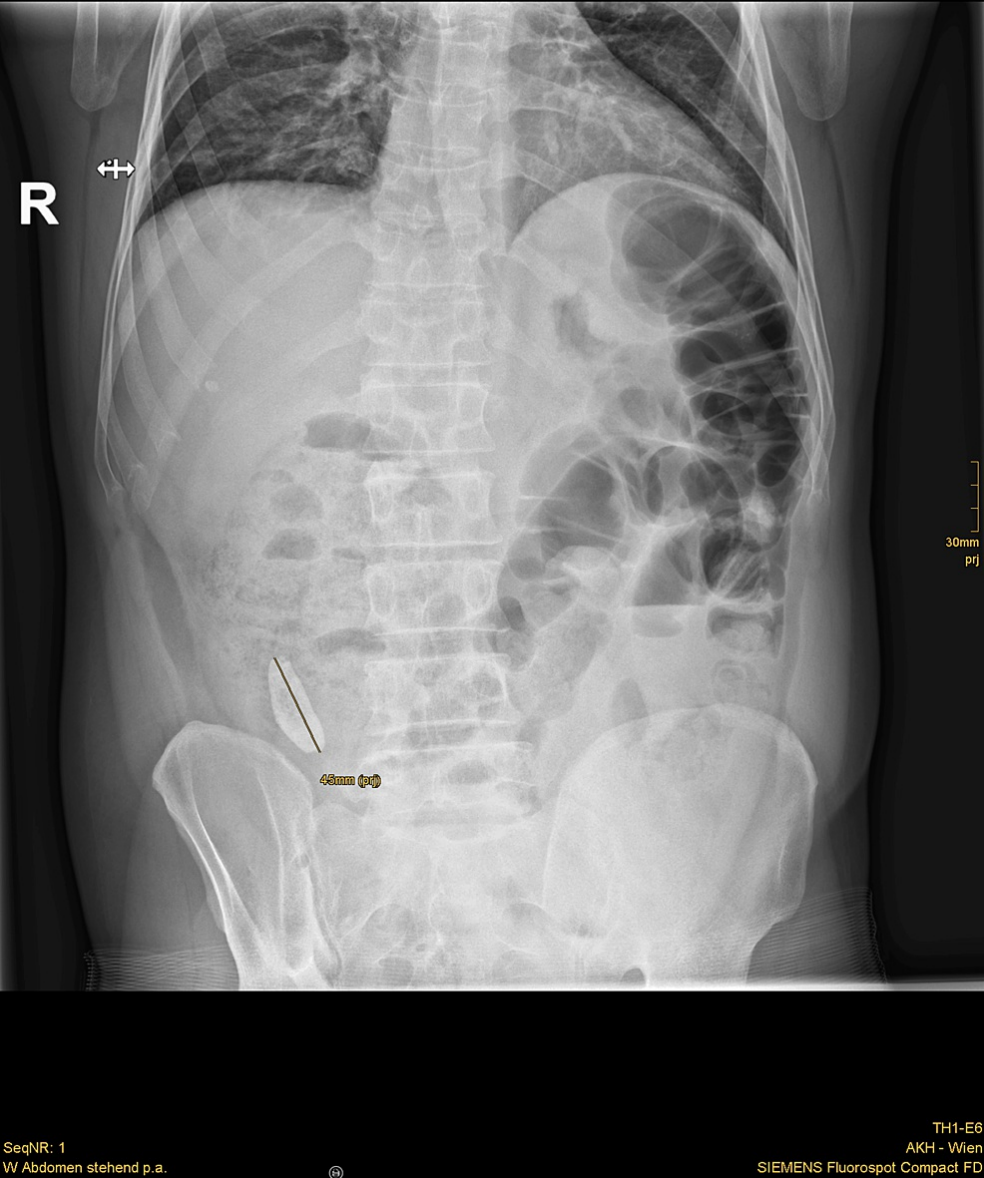
Abdominal X-ray (PA erect view). A dense calcified structure measuring 4.5 cm in the longest diameter is visible in the RLQ. In addition, pathologically distended loops of the small bowel up to 4 cm can be seen in the left abdomen. No pneumoperitoneum is visible, as indicated by the absence of the cupola-sign.

**Figure 2:**
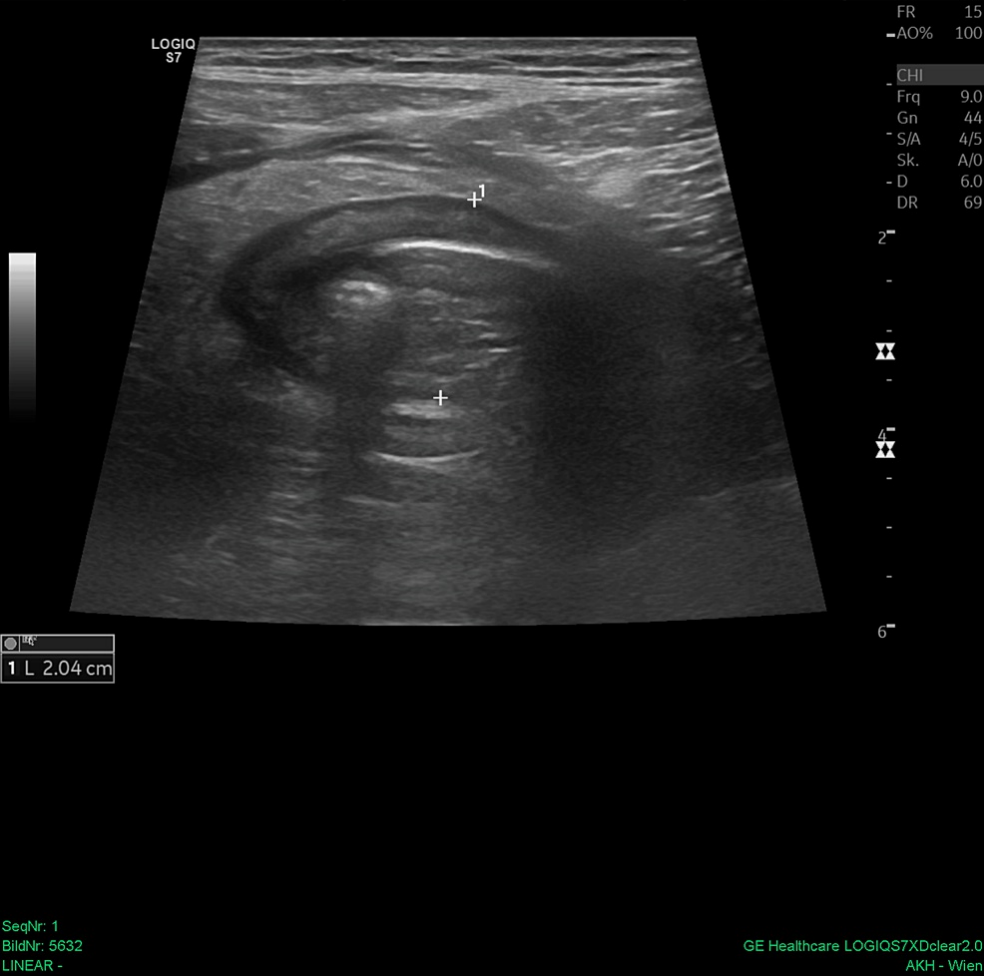
Ultrasound of the right lower quadrant reveals a two-centimeter dilated appendix located at the patient’s point of maximal pain. The appendix contains a hyperechogenic margin with hyperechogenic foci with acoustic shadowing, accompanied by perifocal fat stranding.

**Figure 3:**
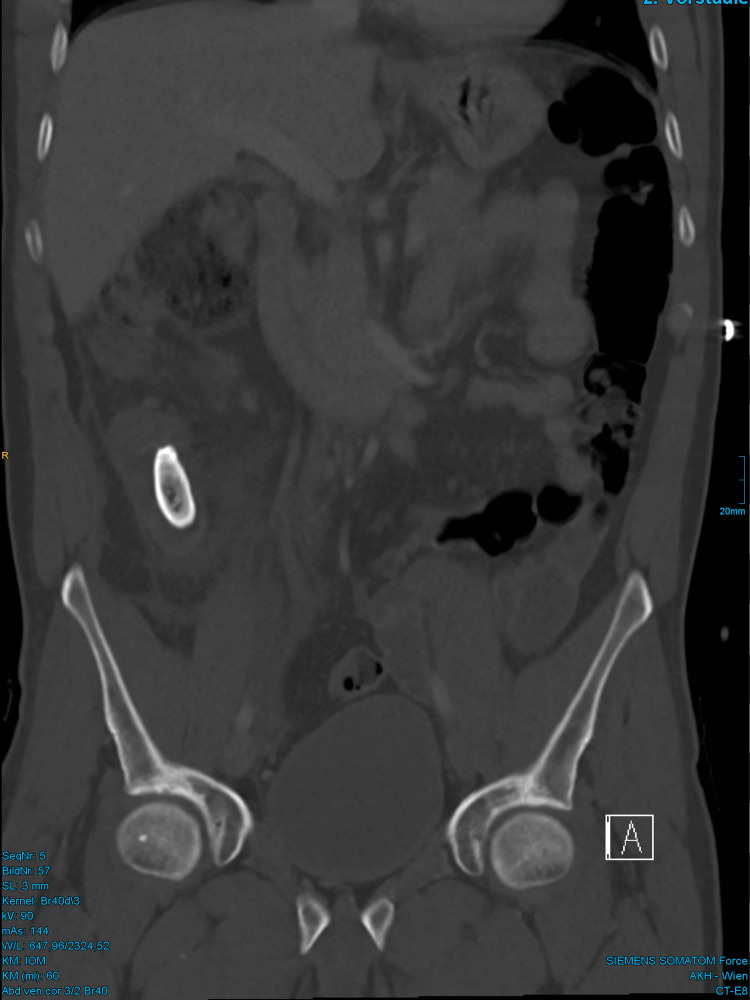
CT scan in portal-venous phase for further pre-operative investigation showing the appendicolith in coronal and axial view (the latter in different grey-level mapping), in concave shape and measuring 4.0 cm × 1.5 cm × 1.9 cm here.

A 44-year-old male patient presented at the surgical outpatient clinic with a distended abdomen and pain, predominantly in the right lower quadrant (RLQ), for three days, along with constipation and the inability to consume solid food. The patient reported no history of prior diseases, surgeries, or medication. Laboratory tests revealed leukocytosis (40 G/L) and elevated CRP levels (18 mg/dL). Abdominal X-rays showed a dense calcified structure measuring 4.5 cm × 1.5 cm in the RLQ, as well as distended loops of the small bowel in the left abdomen (Figure 1). Further imaging with transabdominal ultrasound and an abdominal contrast-enhanced CT scan confirmed the diagnosis of appendicolithiasis, revealing a dilated appendix of up to 2 cm in diameter containing a convex-shaped appendicolith, measuring 4.0 × 1.5 × 1.9 in the CT scan (the reconstructed size of 4.5 cm was depicted), with a small amount of adjacent free fluid and perifocal inflammation of the bowel (Figures 2-3). The latter study was done especially to exclude abscess formation, perforation, pneumoperitoneum, or bowel obstruction and for a more detailed surgical approach.

**Figure 4:**
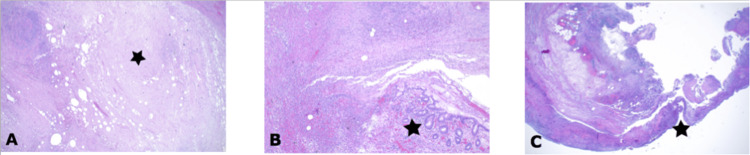
H&E staining of the formalin fixed paraffin embedded tissue showing the homogeneous eosinophilic coprolite (asterisk) in the appendix lumen with heavy inflammation of the surrounding wall (A), ulceration of the mucosa with phlegmonous inflammation and only remnants of crypts left (asterisk, B); perforation (asterisk) due to necrosis of the entire wall with fibrinous and purulent serositis (C).

Considering these findings and after obtaining the patient's consent, the surgical indication for appendectomy was determined. Initially planned as a laparoscopic approach, it was converted to an open surgical procedure via median laparotomy due to the distended bowel, severely inflamed and minimally perforated appendix, and extensive adhesions within the abdomen. After thorough adhesiolysis, the ulcero-phlegmonous appendix containing the appendicolith was removed and sent to histological analysis. Histological analysis confirmed ulcero-phlegmonous appendicolithiasis, periappendicitis, perforation, and serositis (Figure 4). The patient was discharged in stable condition on the seventh postoperative day after managing postoperative bowel paralysis, ongoing abdominal pain, and removing two drains that had been placed in the RLQ and lesser pelvis.

## Discussion

The diagnosis of appendicitis remains clinical, yet laboratory values and radiologic imaging may enhance accuracy, management, and outcome. Transabdominal sonography is the recommended initial imaging strategy, if necessary. A plain abdominal X-ray can be used to investigate the presence of an appendicolith, and a contrast-media-enhanced CT scan can be used for a more precise overview of the presence of possible complications. Pregnant women may benefit from an MRI for further diagnostic evaluation.

## Conclusions

In cases with atypical clinical symptoms or suspected complications, such as suspected peri-appendicitis, abscess, or perforation, a contrast media-enhanced CT scan may provide a more sensitive detection. To the best of our knowledge and according to our research of the literature, this is the largest appendicolith documented, convex-shaped and measuring 4.5 cm.
